# How distributed processing produces false negatives in voxel-based lesion-deficit analyses

**DOI:** 10.1016/j.neuropsychologia.2018.02.025

**Published:** 2018-07-01

**Authors:** Andrea Gajardo-Vidal, Diego L. Lorca-Puls, Jennifer T. Crinion, Jitrachote White, Mohamed L. Seghier, Alex P. Leff, Thomas M.H. Hope, Philipp Ludersdorfer, David W. Green, Howard Bowman, Cathy J. Price

**Affiliations:** a Wellcome Centre for Human Neuroimaging, Institute of Neurology, University College London, London WC1N 3BG, United Kingdom; b Department of Speech, Language and Hearing Sciences, Faculty of Health Sciences, Universidad del Desarrollo, Concepcion 4070001, Chile; c Institute of Cognitive Neuroscience, University College London, London WC1N 3AR, United Kingdom; d Cognitive Neuroimaging Unit, Emirates College for Advanced Education, PO Box 126662, Abu Dhabi, United Arab Emirates; e Department of Brain Repair and Rehabilitation, Institute of Neurology, University College London, London WC1N 3BG, United Kingdom; f Department of Experimental Psychology, Faculty of Brain Sciences, University College London, London WC1H 0AP, United Kingdom; g Centre for Cognitive Neuroscience and Cognitive Systems and the School of Computing, University of Kent, Kent CT2 7NF, United Kingdom; h School of Psychology, University of Birmingham, Birmingham B15 2TT, United Kingdom

**Keywords:** Voxel-based lesion-deficit mapping, Voxel-based morphometry, Voxel-based lesion-symptom mapping, stroke, Anomia, Word-finding difficulties

## Abstract

In this study, we hypothesized that if the same deficit can be caused by damage to one or another part of a distributed neural system, then voxel-based analyses might miss critical lesion sites because preservation of each site will not be consistently associated with preserved function. The first part of our investigation used voxel-based multiple regression analyses of data from 359 right-handed stroke survivors to identify brain regions where lesion load is associated with picture naming abilities after factoring out variance related to object recognition, semantics and speech articulation so as to focus on deficits arising at the word retrieval level. A highly significant lesion-deficit relationship was identified in left temporal and frontal/premotor regions. Post-hoc analyses showed that damage to either of these sites caused the deficit of interest in less than half the affected patients (76/162 = 47%). After excluding all patients with damage to one or both of the identified regions, our second analysis revealed a new region, in the anterior part of the left putamen, which had not been previously detected because many patients had the deficit of interest after temporal or frontal damage that preserved the left putamen. The results illustrate how (i) false negative results arise when the same deficit can be caused by different lesion sites; (ii) some of the missed effects can be unveiled by adopting an iterative approach that systematically excludes patients with lesions to the areas identified in previous analyses, (iii) statistically significant voxel-based lesion-deficit mappings can be driven by a subset of patients; (iv) focal lesions to the identified regions are needed to determine whether the deficit of interest is the consequence of focal damage or much more extensive damage that includes the identified region; and, finally, (v) univariate voxel-based lesion-deficit mappings cannot, in isolation, be used to predict outcome in other patients.

## Highlights

• Post-hoc analyses of results from voxel-based lesion-deficit analyses are reported.
• The effects of different lesion sites cancel each other out when all data are pooled.
• Removing patients with one lesion site can reveal other critical lesion sites.
• Our findings highlight the need for multivariate lesion-deficit analyses.

## 1 Introduction

Mapping lesions to their behavioural consequences remains a key goal in cognitive and clinical neuroscience. In the last decade and a half, the relationship between brain lesion data and behaviour has typically been assessed using mass-univariate techniques such as voxel-based morphometry (VBM; Ashburner and Friston, 2000; Tyler et al., 2005) or voxel-based lesion-symptom mapping (VLSM; Bates et al., 2003; Rorden et al., 2007). These techniques, that we generically describe here as voxel-based lesion-deficit mapping, basically perform thousands of statistical tests on a voxel-by-voxel basis. Voxels that surpass the threshold for statistical significance are then associated with a critical region that, when damaged, causes the deficit of interest. However, despite these advances, voxel-based lesion-deficit mapping studies have also produced inconsistent results in relation to the neural correlates of specific cognitive functions (for a more detailed discussion on this topic, see Inoue et al., 2014; Mah et al., 2014; Price et al., 2017). The goal of the current paper is to disclose and characterise how false negative results can arise in univariate voxel-based lesion-deficit mapping even when the analysis includes data from very large samples of patients. We illustrate this methodological point with a study of the lesion sites that cause word finding difficulties.

Previous voxel-based lesion-deficit mapping studies have shown that the process of word retrieval depends on temporal regions (Baldo et al., 2013, Schwartz et al., 2009), frontal regions (Lacey et al., 2017) and/or the white matter pathways linking temporal and frontal areas in the left hemisphere (Herbet et al., 2016). From a neural network perspective, whereby multiple brain regions contribute to any given cognitive function, it is not surprising that damage to any part of the word retrieval system might impair word finding ability. However, it might be expected that studies that include large samples of patients with a wide variety of lesions and deficits would be more likely to identify all the lesion sites associated with word retrieval ability. The problem we highlight here is that false negatives can arise even if large samples of patients are used because, when a deficit can be caused by damage to more than one region, the lesion-deficit association will be weakened when all subjects are grouped together. For example, if word finding impairments can be a consequence of damage to temporal or frontal regions, a large sample will include patients with no damage to the temporal regions who have word finding difficulties because they have damage to the frontal regions. Likewise, there will be patients with no damage to the frontal regions who have word finding difficulties because they have damage to the temporal regions. The mapping between lesions and deficits will therefore be inconsistent in each region and for each type of deficit, resulting in high unexplained variance and potentially false negative results.

To illustrate how false negative results can arise in univariate voxel-based lesion-deficit analyses, we searched for brain regions where lesion load is associated with word retrieval abilities (as measured by spoken and written picture naming) in hundreds of stroke patients who, collectively, provided a rich sampling of focal or extensive damage to temporal and frontal areas. We then repeated the same analysis, after removing data from patients who had damage to regions identified in the previous step. This increased sensitivity to regions that are associated with the deficit of interest but were not detected in the first analysis because the absence of damage was only rarely associated with preserved function. In addition, we investigated (i) how consistently patients with damage to the identified regions presented with the deficit of interest and (ii) how the results of univariate voxel-based lesion-deficit mapping are influenced by the type of measure (either continuous or binary) used to quantify structural abnormality.

Although we illustrate the utility of iteratively repeating the same lesion-deficit analysis, a full understanding of lesion-deficit relationships will require multivariate methods that take into account how the effect of damage to one region depends on that in another. Recent multivariate approaches to lesion-deficit mapping (e.g. Hope et al., 2013, Hope et al., 2015) include support vector regression (Smith et al., 2013, Forkert et al., 2015, Zhang et al., 2014, Yourganov et al., 2016) and sparse canonical correlation analysis (Pustina et al., 2018). However, despite their relative advantages, sophisticated machine learning techniques are not without problems because (i) as in univariate analyses, they depend on operator decisions such as the definition of a single region (be it a single voxel, an anatomically-defined region or a data-defined region); and (ii) the multi-region lesion information extracted can become very complex and non-intuitive because of the high dimensionality that arises when the same deficit can be caused by damage to multiple regions and, conversely, when multiple deficits are associated with damage to the same region. In this context, established voxel-based approaches are soaring in popularity, because they are still producing novel and interesting information by identifying regions with the most significant lesion-deficit associations and distinguishing the effects of different lesion sites. These data-defined regions could, in future, end up being an important first step for constraining multivariate methods by effectively providing priors for such analyses. Nevertheless, the results of voxel-based analyses should still be interpreted with caution given the complex relationship between lesions and deficits that we illustrate in this paper.

## 2 Materials and methods

### 2.1 Patient selection criteria

Patients were selected from the Predicting Language Outcome and Recovery After Stroke (PLORAS) database (Seghier et al., 2016) according to the following criteria: (i) left-hemisphere stroke attested by a clinical neurologist and defined by an automated lesion identification algorithm (Seghier et al., 2008); (ii) patients with lesions larger than 1 cm^3^; (iii) native speakers of English; (iv) right-handed prior to their stroke; and, (v) more than 3 months since stroke. These criteria were met by 359 left-hemisphere stroke patients, aged between 21 and 90 (mean age 59.4). Table 1 provides demographic and behavioural information for all participants. All individuals had undergone language testing on the Comprehensive Aphasia Test (Swinburn et al., 2004) and high-resolution structural MRI scanning. The study was approved by the London Queen Square Research Ethics Committee. All patients gave written informed consent prior to participation and were compensated £ 10 per hour for their time.

**Table 1:** Summary of demographic and clinical data for Analyses 1, 2 and 3.

		**Initial Sample**	**With Fuzzy Images**		**With Binary Images**	
**Factor**		**Analysis 1 (n = 359)**	**Analysis 2 (n = 127)**	**Analysis 3 (n = 114)**	**Analysis 2 (n = 144)**	**Analysis 3 (n = 118)**
Age at stroke onset (years)	*M (SD)*	59.4 (12.4)	57.6 (13.2)	58.6 (13.0)	57.4 (12.6)	58.5 (12.6)
	Range	21.3 − 90.0	22.8 − 85.9	24.9 − 85.9	22.8 − 85.9	24.9 − 85.9
Age at testing (years)	*M (SD)*	54.4 (12.9)	61.2 (13.6)	62.2 (13.3)	61.1 (13.0)	62.1 (13.1)
	Range	17.2 − 86.5	23.1 − 87.4	26.5 − 87.4	23.1 − 87.4	26.5 − 87.4
Time post-stroke (years)	*M (SD)*	5.0 (5.2)	3.6 (3.6)	3.6 (3.5)	3.8 (3.6)	3.5 (3.4)
	Range	0.3 − 36.0	0.3 − 19.5	0.3 − 19.5	0.3 − 19.5	0.3 − 19.5
Education (years)	*M (SD)*	14.5 (3.2)	15 (3.8)	15.1 (3.8)	14.9 (3.7)	15.1 (3.8)
	Range	10 − 30	11 − 30	11 − 30	10 − 30	11 − 30
Lesion volume (cm^3^)	*M (SD)*	85.7 (87.7)	15.9 (16.3)	15.5 (16.5)	20.0 (22.0)	16.3 (19.6)
	Range	1.2 − 386.2	1.2 − 93.1	1.2 − 93.1	1.2 − 119.2	1.2 − 119.2
Gender	Males	248	85	78	96	84
	Females	111	42	36	48	34
Spk-PN	Imp/Non	192/167	32/95	27/87	39/105	26/92
	*M (SD)*	59.9 (10.5)	66.7 (7.5)	67.3 (7.0)	66.4 (7.6)	67.5 (7.0)
Writt-PN	Imp/Non	102/257	10/117	7/107	11/133	6/112
	*M (SD)*	58.7 (8.6)	63.5 (5.4)	63.9 (4.9)	63.5 (5.4)	64.2 (4.7)
Rep-N	Imp/Non	129/230	25/102	23/91	31/113	23/95
	*M (SD)*	54.6 (9.1)	58.8 (7.8)	58.8 (7.8)	58.4 (8.0)	58.5 (7.8)
Sem-M	Imp/Non	35/324	8/119	7/107	9/135	7/111
	*M (SD)*	56.6 (6.1)	57.4 (4.8)	57.4 (4.9)	57.4 (5.0)	57.6 (4.8)
CSpk-W	Imp/Non	75/284	16/111	14/100	18/126	13/105
	*M (SD)*	57.1 (6.8)	59.4 (5.8)	59.5 (5.8)	59.2 (5.0)	59.5 (5.8)
Writt-Copy	Imp/Non	43/316	4/123	3/111	6/138	4/114
	*M (SD)*	58.4 (5.4)	60.0 (3.1)	60.1 (2.9)	59.8 (3.4)	60.0 (3.1)

Patients in Analyses 2 and 3 were subsets of the full sample of 359 left-hemisphere stroke patients from Analysis 1 (see Section 2).

**Abbreviations:** M = mean across groups; SD = standard deviation; Spk-PN = spoken picture name; Writt-PN = written picture name; Rep-N = repetition of nonwords; Sem-M = semantic memory; CSpk-W = spoken word comprehension; Writt-Copy = written copy; Imp/Non = Impaired/Non-impaired performance.

### 2.2 Behavioural assessment

All 359 patients were assessed with 27 tasks from the Comprehensive Aphasia Test (CAT; Swinburn et al., 2004). The total score for every assessment is converted into a T-score, which represents how well the patient performed relative to a reference population of 113 aphasic patients, 56 of whom were tested more than once on the CAT. The threshold for impairment is defined relative to a second reference population of 27 neurologically-normal controls, as the point below which the score would place the patient in the bottom 5% of the control population (for more details on the standardisation samples, see Swinburn et al., 2004).

As we were primarily interested in identifying brain regions where damage was associated with word retrieval deficits, we only focus on the results of 6 tasks. Two tasks tested the patients’ ability to retrieve and name pictures of objects in two different modalities; these were the spoken and written picture naming tasks with a word retrieval impairment expected to affect performance on both tasks. Additionally, in order to focus on word retrieval deficits, we included four tasks that tested the patient's ability to: (1) articulate (i.e. motor control of speech) using nonword repetition; (2) recognise, process and remember the semantic content of pictures and auditory words using semantic associations and spoken word comprehension; and, (3) control hand movements using letter and word copying. Task details were as follows:

#### 2.2.1 Task 1

The CAT spoken picture naming (Spk-PN) task visually presents 24 line drawing pictures of objects (e.g., knife), one at a time, with instructions to name them aloud. Articulatory errors (e.g., dysarthric distortions) not affecting the perceptual identity of the target were scored as correct. Verbal, phonemic, neologistic and apraxic errors were scored as incorrect. T-scores equal to or below 61 constitute the impaired range.

#### 2.2.2 Task 2

The CAT written picture naming (Writt-PN) task visually presents five pictures of objects (e.g., tank), one at a time, with instructions to write their names down. Letters in the correct position were given a score of 1 each. Substitutions, omissions and transpositions were given a score of 0. One point was deducted from the total score if one or more letters were added to the target word. T-scores equal to or below 54 constitute the impaired range.

#### 2.2.3 Task 3

The CAT nonword repetition (Rep-N) task presents five nonsense words (e.g., gart), one at a time, with instructions to repeat them aloud. Immediate correct responses were given a score of 2; incorrect responses were given a score of 0; correct responses after a self-correction or a delay (> 5 s) were given a score of 1. Articulatory errors (e.g., dysarthric distortions) not affecting the perceptual identity of the target were scored as correct. Verbal, phonemic, neologistic and apraxic errors were scored as incorrect. T-scores equal to or below 52 constitute the impaired range.

#### 2.2.4 Task 4

The CAT writing copy (Writt-Copy) task visually presents letters and words that the participant is prompted to copy: five letters from upper to upper case and five letters from lower to upper case. Additionally, the patient is asked to copy three words using only capital letters. Correct responses were given a score of 1; incorrect responses were given a score of 0. T-scores equal to or below 51 constitute the impaired range.

#### 2.2.5 Task 5

The CAT spoken word comprehension (CSpok-W) task involves hearing a word produced by the examiner and selecting the picture among four possible alternatives that best matches the meaning of the heard word. There are a total of fifteen test trials plus a practice one at the beginning. The scoring system for this task was identical to that used in the nonword reading task. T-scores equal to or below 52 constitute the impaired range.

#### 2.2.6 Task 6

The CAT semantic associations (Sem-M) task visually presents five pictures of objects simultaneously on each trial. The instructions are to match the picture at the centre (e.g., mitten) with one of four possible alternatives according to the strongest semantic association (e.g., hand, sock, jersey, and lighthouse). The inclusion of a semantically related distractor (e.g., sock) encouraged deeper levels of semantic processing. There are a total of ten test trials plus a practice one at the beginning. Correct responses were given a score of 1; incorrect responses were given a score of 0. T-scores equal to or below 50 constitute the impaired range.

### 2.3 MRI data acquisition, pre-processing and lesion identification

T1-weighted high resolution anatomical whole-brain volumes were available for all patients (n = 359). Four different MRI scanners (Siemens Healthcare, Erlangen, Germany) were used to acquire the structural images: 167 patients were imaged on a 3 T Trio scanner, 130 on a 1.5 T Sonata scanner, 57 on a 1.5 T Avanto scanner, and five on a 3 T Allegra scanner. Each of these T1-weighted images was then submitted to our fully automated lesion identification procedure for lesion detection and delineation (see below for details). This converts a scanner-sensitive raw image into a quantitative assessment of structural abnormality that should be independent of the scanner used. Additionally, the quality of the generated lesion images is evaluated by visually inspecting the results. Three types of lesion identification errors, which might differ from manually drawn lesions, have been detected. First, the lesion extent includes cerebrospinal fluid in enlarged ventricles. Second, cortical atrophy, e.g. around the dorsal parietal lobes can sometimes be included in the lesion image. Third, the automated approach can miss small cortical lesions where there is normal inter-subject variability in sulci. In addition, there are potential errors that arise in both automated and manually defined lesions, particularly in the specification of the border of the lesion which is typically gradual rather than categorical. We did not attempt to correct any of these errors (none of our lesions were manually drawn) and they therefore increased “noise” in the analysis, which may have resulted in false negatives but did not result in false positives as confirmed by our post-hoc analyses. However, we did remove an artefact that was identified as a lesion (in the absence of damage) in the brain stem for several of the patients who were scanned on the 1.5 T Avanto scanner.

For anatomical images acquired on the 1.5 T Avanto scanner, a 3D magnetization-prepared rapid acquisition gradient-echo (MPRAGE) sequence was used to acquire 176 sagittal slices with a matrix size of 256 × 224, yielding a final spatial resolution of 1 mm isotropic voxels (repetition time/echo time/inversion time = 2730/3.57/1000 ms). For anatomical images acquired on the other three scanners, an optimised 3D modified driven equilibrium Fourier transform (MDEFT) sequence was used to acquire 176 sagittal slices with a matrix size of 256 × 224, yielding a final spatial resolution of 1 mm isotropic voxels: repetition time/echo time/inversion time = 12.24/3.56/530 ms and 7.92/2.48/910 ms at 1.5 T and 3 T, respectively (Deichmann et al., 2004).

All T1-weighted images were converted to 3D lesion images in standard MNI space as described in Seghier et al. (2008). Two types of 3D lesion images were obtained from our automated lesion identification procedure: (i) a fuzzy lesion image encoding the degree of abnormality on a continuous scale from 0 (completely normal) to 1 (completely abnormal) at each given voxel relative to normative data drawn from a sample of 64 neurologically-normal controls; and (ii) a binary lesion image, which is simply a thresholded (i.e. lesion/no lesion) version of the fuzzy lesion image – used here to delineate the lesions, to estimate lesion volume, and to generate lesion overlap maps. The threshold used to convert the fuzzy into binary lesion images was 0.3 as recommended in Seghier et al. (2008). Importantly, the *U* value (i.e. > 0.3) has been optimised to obtain the most accurate results from data collected on our scanners. The binary lesion images were also used to investigate how the results of our regression model changed with binary versus continuous (fuzzy) measures of structural abnormality; see Table 2, Table 3.

**Table 2:** Brain regions identified by voxel-based lesion-deficit analyses.

**A. Fuzzy images, first iteration (n = 359)**				**voxel-level**		**cluster-level**	
**Region**	**x**	**y**	**z**	**Z**_**score**_	**P**_**FWE-corr**_	**voxels**	**P**_**FWE-corr**_
Left Middle Temporal Lobe	−40	−32	2	4.96	0.002	280	0.000
Left Inferior Frontal Cortex	−44	0	18	4.62	0.010	35	0.011
*Post-hoc analysis of regions identified in Analysis 2, lowering statistical threshold to p < .05 uncorrected.*							
*Left Anterior Putamen*	*−20*	*10*	*2*	*2.26*	–	–	–
**B. Fuzzy images, second iteration (n = 127)**							
Left Anterior Putamen	−20	10	2	3.91	0.045	17*	0.041
**C. Binary images, first iteration (n = 359)**							
Left Middle Temporal Lobe	−42	−46	10	5.16	0.005	40	0.002
Left Inferior Frontal Cortex	−44	2	20	4.94	0.014	8	0.004
*Post-hoc analysis of regions identified in Analysis 2, lowering statistical threshold to p < .05* uncorrected.							
*Left Anterior Putamen*	*−18*	*12*	*−2*	*2.54*	–	–	–
**D. Binary images, second iteration (n = 144)**							
Left Anterior Putamen	−18	12	−2	5.41	0.000	254*	0.000

This table shows all clusters/areas where lesion load was significantly correlated with word finding abilities. All regions listed below were in the left hemisphere and the coordinates reported in MNI space; x y z = MNI coordinates; P_FWE-corr_ = p-value corrected (family-wise error correction) for multiple comparisons; P_uncorr_ = p-value uncorrected. * = number of voxels that survived a voxel-level threshold of p < 0.001 uncorrected.

**Table 3:** Number of patients with impairments after damage to each ROI.

**A. Regions from first iteration analyses**									
**Degree of damage to each ROI**			**Analysis with Fuzzy images**			**Analysis with Binary images**			
**Group**	**Temporal ROI**	**Frontal ROI**	**n**	**Impaired**	**Not impaired**	**n**		**Impaired**	**Not impaired**
**Frontal (1)**	≤ 25%	≥ 75%	68	18	50	77		19	58
**Temporal (2)**	≥ 75%	≤ 25%	23	10	13	25		11	14
**Both (3)**	≥ 75%	≥ 75%	71	48	23	63		45	18
**Partial (4)**	74%−26%	74%−26%	70	13	57	50		13	37
**Neither (5)**	≤ 25%	≤ 25%	127	9	118	144		10	134
**B. Regions from second iteration analyses**									
			**Analysis with Fuzzy images**				**Analysis with Binary images**		
**Degree of damage of Putamen ROI**			**n**	**Impaired**	**Not Impaired**	**n**		**Impaired**	**Not Impaired**
Putamen damaged (> 25%)			13	3	10	26		5	21
Putamen preserved (**≤** 25%)			114	6	108	118		5	113

The table shows the number of patients who had impairments (or did not meet the criteria for impairments; see Section 2). Group = patients were assigned to one of five different groups (i.e. numbers in brackets) according to the degree of damage they had incurred to the regions identified in our voxel-based lesion-deficit analyses. ROI = region of interest; n = number of patients; Impaired = number of patients with the deficit of interest (i.e. impaired performance on the spoken and written picture naming tasks); Not impaired = number of patients who did not have the deficit of interest.

### 2.4 Lesion-deficit analyses

We used voxel-based morphometry (VBM; Ashburner and Friston, 2000) to assess lesion-deficit relationships (Tyler et al., 2005), performed in SPM12 using the general linear model. The goal of all statistical analyses was to identify brain regions where lesion load is significantly associated with word retrieval abilities (as measured by both spoken and written picture naming). The imaging data entered into each analyses were either the fuzzy or binary images that are produced by our automated lesion identification toolbox (see above).

The most important advantage of utilising the fuzzy lesion images (as in Price et al., 2010) over alternative methods is that they provide a quantitative measure of the degree of structural abnormality, at each and every voxel of the brain, relative to neurologically-normal controls. In contrast to fuzzy lesion images, (i) binary lesion images do not provide a continuous measure of structural abnormality and will be less sensitive to subtle changes that are below an arbitrary threshold for damage (see Fridriksson et al., 2013); and (ii) segmented grey or white matter probability images when used in isolation (as in standard VBM routines) do not provide a complete account of the whole of the lesion (e.g., Mehta et al., 2003). By repeating exactly the same analyses with either the fuzzy or binary images, we are able to measure the sensitivity of each.

**Fig. 1:**
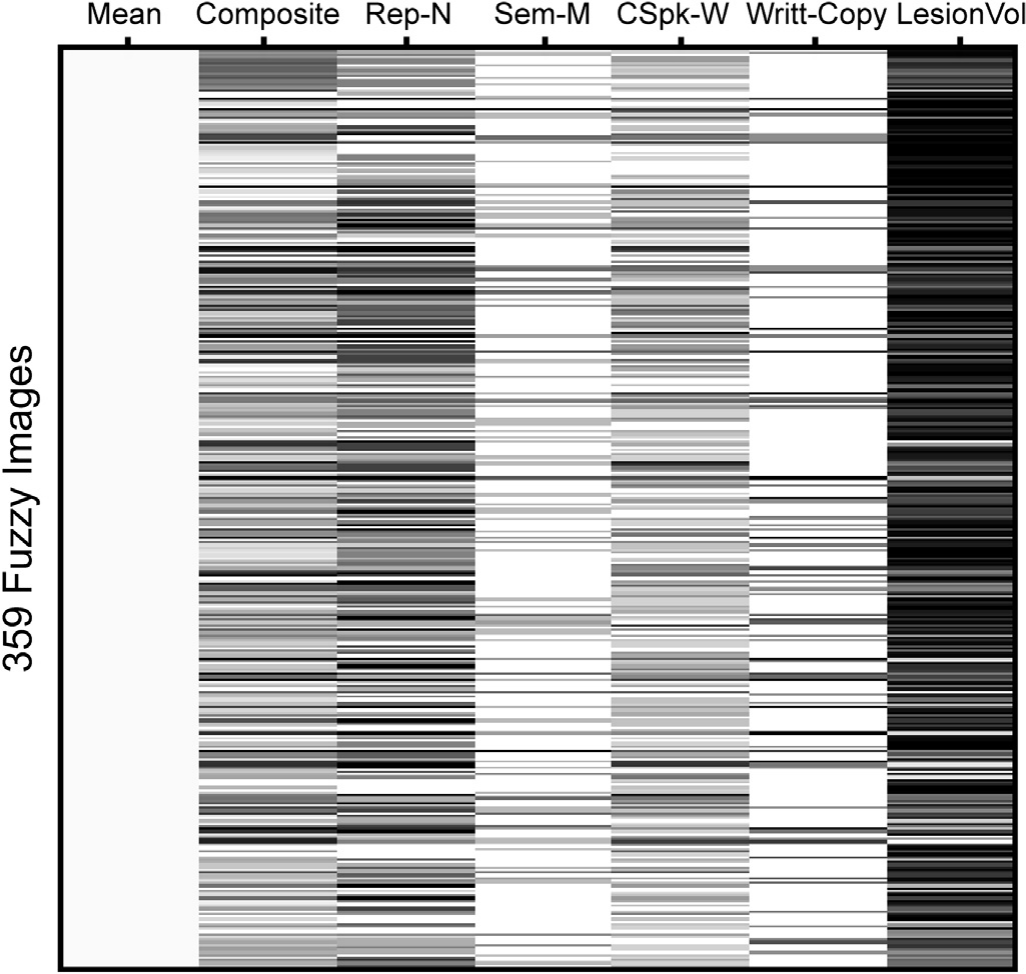
Design matrix for Analysis 1. The figure shows the design matrix for the multiple regression model (6 regressors) from Analysis 1. The same design matrix was used for Analyses 2 and 3 (see Section 2). Composite score (i.e. Composite) was the average scores of the spoken and written picture naming tasks. *See Table 1 for abbreviations.

In Analysis 1 (which included data from all 359 participants), the fuzzy (or binary) lesion images were entered into a multiple regression model with 6 different regressors (5 behavioural scores and lesion size, see Fig. 1). The regressor of interest was the average of the scores of Tasks 1 and 2 (spoken picture naming and written picture naming), both of which are sensitive to word retrieval abilities (labelled ‘composite score’ in Fig. 1). Patients who had impairments on both tasks had the lowest scores on this composite score but it was also theoretically possible for low scores to be a consequence of a severe impairment on one picture naming task but not the other. Additionally, we included regressors of no interest to factor out variance related to speech production (using nonword repetition scores), semantic picture matching (using semantic associations scores), speech comprehension (using spoken word comprehension scores); and hand writing (using letter and word copying scores), along with lesion size. Regions of interest were those where a significant lesion-deficit relationship was observed after family-wise error (FWE) correction for multiple comparisons (estimated using random field theory as implemented in SPM; for more details, see Flandin and Friston, 2015) across the whole search volume. Finally, we conducted post-hoc tests to verify that damage to the identified regions explained all the patients with word finding difficulties.

In Analysis 2, we tested whether additional lesion-deficit associations could be identified. To this end, we repeated Analysis 1 after excluding all patients with more than 25% damage to the region(s) identified in Analysis 1. In Analysis 3, we repeated the same procedure again after excluding all patients who had more than 25% damage to the region(s) identified in Analyses 1 and 2. In other words, we continually (and deliberately) biased our patient selection to those who did not have damage to regions identified in previous steps. By definition, each iteration is conducted with smaller and smaller numbers of patients until no further voxels survive FWE-corrected p thresholds (estimated using random field theory as implemented in SPM). In addition, we performed a set of analyses on synthetically-generated null data sets to test the statistical robustness of the core principles upon which our iterative procedure was built. The simulations suggest that the procedure does not inflate the false positive rate. For a more detailed explanation and full disclosure of results, please see Supplementary material.

**Fig. 2:**
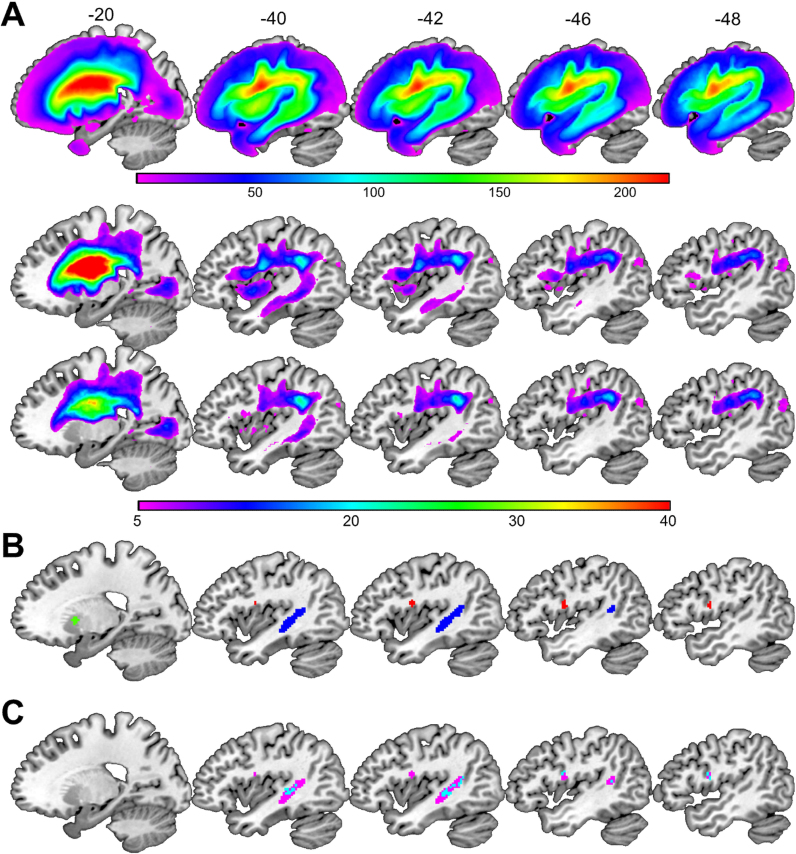
Lesion overlap maps and regions identified in Analyses 1 and 2. (A) From top to bottom, the lesion overlap maps for Analysis 1 (n = 359), Analysis 2 (n = 127) and Analysis 3 (n = 114) are shown in sagittal slices. The colour scale indicates the number of patients with overlapping lesions at each given voxel. (B) The regions identified in Analysis 1 (with fuzzy images) are highlighted in blue (the temporal region) and red (the frontal region). The region identified in Analysis 2 (the putamen) is highlighted in green. The temporal and frontal regions are thresholded at p < 0.05 FWE-corrected. The putamen region is thresholded at p < 0.001 uncorrected for visualization purposes only. (C) The significant lesion-deficit associations identified in Analysis 1 using the: (i) fuzzy lesion images are shown in pink; (ii) binary lesion images are shown in blue. Cyan is the overlap. (For interpretation of the references to color in this figure legend, the reader is referred to the web version of this article.).

The search volume was limited to voxels that were damaged in at least five patients from the group being examined. For this purpose, lesion overlap maps based on the binary lesion images were created for each analysis (see Fig. 2A), thresholded at five, and used as inclusive masks before estimating the model. Our voxel-level statistical threshold was set at p < 0.05 after family-wise error correction for multiple comparisons (estimated using random field theory as implemented in SPM; for alternative multiple comparison correction approaches, see Mirman et al., 2018) across the whole search volume; see Table 2.

## 3 Results

### 3.1 Analysis 1: full sample of 359 stroke patients

Using the fuzzy images, our deficit of interest was associated with greater lesion load in two spatially distinct clusters. The first cluster (280 voxels in size) was in the left temporal lobe extending from the posterior middle temporal gyrus into the arcuate fasciculus, temporal stem and anterior superior temporal gyrus. The second cluster (35 voxels in size) was centred on the left ventral premotor cortex extending into the opercular part of the inferior frontal gyrus. For brevity we refer to these regions, henceforth, as the temporal and frontal regions. When the analysis was replicated using the binary instead of the fuzzy lesion images, the same anatomical regions were identified (overlap = 93% for the temporal region and 75% for the frontal region; see Fig. 2C). The only noticeable difference between both analyses was in the extent of the effect: more voxels surpassed the threshold for statistical significance when the fuzzy lesion images were used (see Table 2).

For the post-hoc analyses, we assigned each of the 359 patients to one of five different groups according to how much damage they had incurred to the temporal and frontal regions identified in Analysis 1 (see Table 3). Group 1 had 75–100% damage to the frontal region with 0–25% damage to the temporal region (n = 68); Group 2 had 75–100% damage to the temporal region with 0–25% damage to the frontal region (n = 23); Group 3 had 75–100% damage to both regions (n = 71); Group 4 included all patients with 26–74% damage to one or both regions (n = 70); and Group 5 had 0–25% damage to both regions (n = 127). This grouping strategy aimed to maximise differences in lesion sites between groups.

Having assigned the patients to different groups, we compared the incidence and severity of our deficit of interest (written and spoken picture naming) across groups (see Table 3). In terms of the incidence, we found that the deficit of interest (impaired spoken and written picture naming) was observed in 18/68 patients (26%) with ≥ 75% damage to the frontal region (Group 1), 10/23 patients (43%) with ≥ 75% damage to the temporal region (Group 2), 48/71 patients (68%) with ≥ 75% damage to the temporal and frontal regions (Group 3), 13/70 (19%) patients with 24–74% damage to both regions (Group 4), and 9/127 (7%) of those with 0–25% damage to both regions (Group 5). Therefore, the incidence of the deficit of interest was significantly higher in Groups 1 (*X*^*2*^ = 13.95, p < 0.001), 2 (*X*^*2*^ = 23.3, p < 0.001), and 3 (*X*^*2*^ = 81.4, p < 0.001) compared to Group 5 (i.e. those with relative sparing of the temporal and frontal regions).

In terms of the severity, the mean spoken and written picture naming scores were significantly worse in patients who had substantial damage to either or both of the identified regions (Groups 1, 2 and 3) relative to those who had relative sparing of both regions (Group 5; p < 0.001, Bonferroni-corrected for the number of pairwise comparisons), even after accounting for the effect of lesion size (*F*(4353) = 15.70, p < 0.001). However, there were no significant differences (p = 1.000) in picture naming scores for those with frontal versus temporal lesions (Groups 1 versus 2).

Although our analysis has identified regions that are damaged in most patients with the deficit of interest (i.e. deficit-to-lesion mapping), it was not the case that damage to each of the identified areas consistently caused the deficit of interest (i.e. lesion-to-deficit mapping). Indeed, there were less patients with, than without, the deficit of interest when only the temporal region or only the frontal region was damaged (Groups 1 and 2); see Table 3. This indicates that statistically significant lesion-deficit mappings can be driven by only a subset of the patients in the sample.

Examination of the lesion sites for patients within Groups 1 and 2 highlighted another point that is relevant for interpreting the results from the lesion-deficit mapping: all the patients with the deficit of interest (n = 28) had lesions which were much larger than the regions that reached statistical significance in the voxel-based analysis. We therefore have no evidence to suggest that focal damage to either the temporal or frontal regions (i.e. sparing other surrounding areas) is sufficient to cause the deficit of interest. For example, in Group 2 patients with the deficit of interest (i.e. impaired performance on both the spoken and written picture naming tasks) had very large lesions (> 40.1 cm^3^) that extended outside the borders of our temporal region. Likewise, in Group 1, the smallest lesion associated with the deficit of interest was 7.6 cm^3^ and included the inferior and middle frontal gyrus, and superior parts of the insula (see Fig. 4).

In summary, by mapping deficit-to-lesion, Analysis 1 identified two regions (temporal and frontal) that were partially or substantially damaged in 91% (89/98) of the patients with the deficit of interest. We also found that (i) the process of interest was not more or less affected by damage to the temporal region or the frontal region; (ii) damage to either region was associated with the deficit of interest in less than 50% of the patients; and (iii) the lesions in those with word finding difficulties extended beyond the boundaries of the temporal and frontal regions derived from Analysis 1. As our sample of 359 patients did not include any patients with selective damage to one of the identified regions (sparing surrounding areas), our data do not indicate whether focal lesions to either the temporal or frontal regions is sufficient to cause word finding difficulties. Nor have we explained why partial damage to one or both of our regions caused word finding difficulties in some patients but not in others (Group 4).

### 3.2 Analysis 2: subsample of 127 stroke patients with 0–25% damage to the temporal and frontal regions

**Fig. 3:**
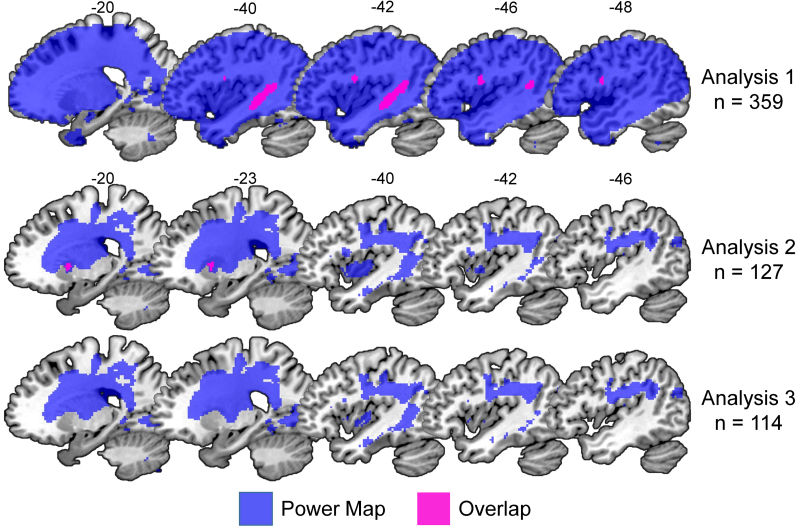
Maps of statistical power. The brain regions coloured in blue indicate sufficient statistical power to detect a significant lesion-deficit association at a threshold of *p* < 0.05 after correction for multiple comparisons. The top two rows illustrate the significant lesion-deficit association and power map for Analyses 1 and 2. Pink is the overlap (= 100%). The bottom row illustrates the power map for Analysis 3 (n = 114), which did not yield any significant effects. The statistical power maps were generated using the “nii_powermap” function of NiiStat (https://www.nitrc.org/projects/niistat/), which is a set of Matlab scripts for analysing neuroimaging data from clinical populations. (For interpretation of the references to color in this figure legend, the reader is referred to the web version of this article.).

After removing 232 patients who had 26–100% damage to the temporal and/or frontal regions. Analysis 2 found a significant lesion-deficit association in the left anterior putamen (see green and pink clusters in Figs. 2B and 3) that was not identified in Analysis 1, even when lowering the threshold to p < 0.001 uncorrected, and despite Analysis 1 including all the patient data used in Analysis 2.

The significance of the effect in Analysis 2 cannot be explained in terms of an inflation of the false positive rate as a result of repeating the voxel-based lesion-deficit mapping because, theoretically, Analysis 2 should have less statistical power than Analysis 1 (see Fig. 3), after excluding data from many patients (n = 53) who had severe word finding difficulties and putamen lesions that co-occurred with damage to the temporal and/or frontal regions identified in Analysis 1. Instead, the discrepancy between the analyses can be explained by the fact that 37 patients in Analysis 1 had the deficit of interest in the context of minimum damage to the left putamen (0–25%). This would have created inconsistency in the lesion-deficit association in the left putamen (many patients had the deficit but no lesion to the putamen). Removing most of these patients (n = 31), therefore, increased the proportion of patients with intact putamen and preserved word finding abilities, which in turn sensitised Analysis 2 to the association of putamen lesions with the deficit of interest.

**Fig. 4:**
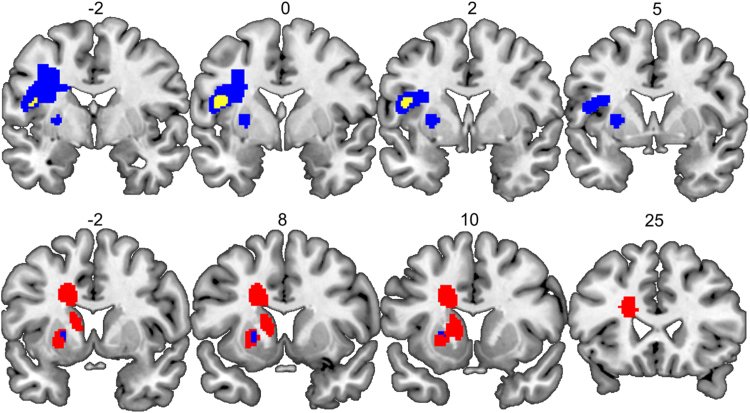
An illustration that the smallest lesion sites were bigger than the regions identified in each analysis. **Top row:** The frontal region identified in Analysis 1 is shown in yellow, and the smallest lesion site associated with word finding difficulties following damage to the frontal region is shown in blue. **Bottom row:** The putamen region identified in Analysis 2 is shown in blue, and the smallest lesion site associated with word finding difficulties following damage to the putamen region is shown in red. Numbers above indicate x coordinates of the coronal slices in MNI space. (For interpretation of the references to color in this figure legend, the reader is referred to the web version of this article.).

Post-hoc analyses showed that the putamen region was damaged (26–100%) in 13/127 patients in Analysis 2 (see Table 2, Table 3) and 3/13 of these patients had impaired picture naming scores. These 3 patients illustrate that Analysis 2 has successfully identified lesion sites that were not associated with word finding difficulties in Analysis 1. However, we also note that (i) as in Analysis 1, the lesion-deficit relationship was driven by a small subset of subjects and (ii) the smallest putamen lesion associated with word finding difficulties was 20 cm^3^ and comprised different brain areas fed by the left middle/posterior cerebral artery including the caudate and surrounding white matter, the lentiform nucleus; and parts of the thalamus (see Fig. 4). The point being that, although the lesion-deficit association was most significant in the left anterior putamen, there were no patients with focal damage to this region. We are therefore not able to conclude that left putamen damage alone can cause word finding difficulties (just as we are not able to conclude that focal lesions to the areas identified in Analysis 1 can cause word finding difficulties).

### 3.3 Analysis 3: subsample of 114 stroke patients with 0–25% damage to the regions identified in Analyses 1 and 2

The third analysis did not yield any significant voxels (i.e. p < 0.05, FWE-corrected; see Fig. 3), and thus our iterative lesion-deficit mapping stopped here.

## 4 Discussion

Mass-univariate voxel-based lesion-deficit mapping (VBM or VLSM) typically involves searching the whole brain for regions where there is a statistically significant association between damage and a deficit of interest in the patient sample being investigated. The current study illustrates some of the limitations of this approach by conducting post-hoc analyses that report (a) the incidence and severity of impairments in those with damage to the identified regions and (b) new regions that are detected when the voxel-based search is repeated after excluding all patients with damage to the regions identified in the preceding analysis. We have defined the deficit of interest as word finding difficulties, however, the point of the paper is not to describe the brain regions where damage causes word finding difficulties but to highlight the challenges in drawing such conclusions.

Below we discuss how (i) the results of voxel-based lesion analyses are affected when the same deficit can be caused by different lesion sites (i.e. distributed processing); (ii) highly significant effects can be observed in regions where damage impairs the function of interest in only a small subset of the patients; (iii) damage limited to regions identified by voxel-based lesion analyses may not be sufficient to cause the deficit of interest; and (iv) the pros and cons of conducting voxel-based lesion analyses on binary (indexing the presence or absence of damage) compared to continuous measures of structural abnormality. Finally, (v) we consider the implications of these findings for future lesion-deficit studies.

### 4.1 When the same deficit can be caused by different lesion sites

Our voxel-based lesion-deficit mapping analyses identified three different lesions sites associated with the deficit of interest; Analysis 1 yielded two anatomically distinct lesion sites in frontal and temporal regions that have been reported in previous studies of word finding difficulties (DeLeon et al., 2007, Cloutman et al., 2009, Baldo et al., 2013, Lacey et al., 2017). Analysis 2 revealed a third region in the left anterior putamen that did not reach statistical significance in Analysis 1. The identification of a new region in Analysis 2 illustrates that univariate voxel-based lesion-deficit analyses that pool over participants (as in Analysis 1) might not detect some or all of the lesion-deficit mappings when the same deficit can be the consequence of multiple different lesion sites. For example, in our case, the association between damage to the left putamen and the deficit of interest was compromised in Analysis 1 because there were 37 patients who did not have damage to the left putamen but did have word finding difficulties as a result of damage to other regions (e.g. frontal and/or temporal). Most of these patients were removed from Analysis 2 thereby strengthening the relationship between the presence/absence of damage to the left putamen and the presence/absence of the deficit of interest.

### 4.2 Significant effects where damage only causes a deficit in a small subset of patients

Our findings highlight that statistically significant lesion-deficit associations in group level studies can be driven by only a small subset of patients. For example, although Analysis 1 identified a highly significant relationship between the deficit of interest (i.e. word retrieval difficulties) and lesion load in two anatomically distinct regions in the frontal and temporal lobes, post-hoc analyses revealed that, from the total sample of 359 patients, only 68 subjects had substantial damage to the frontal region, 23 to the temporal region, and 71 to both the temporal and frontal regions. More importantly, when we calculated the number of patients with substantial damage to either of the regions, we observed that less than half of them (26% and 43%, respectively) had the deficit of interest (see Table 3). Similarly, in our second analysis (n = 127), post-hoc tests revealed, once again, that the mapping between lesion site and deficit was inconsistent in the third region (in the left anterior putamen): 13 patients had damage to the putamen region, however, only 3 of them had the deficit of interest. Critically, when these 3 patients with damage to the putamen and the deficit of interest were removed from the analysis, we could no longer find any significant lesion-deficit associations (see Results).

These post-hoc observations allow us to make two points. First, they demonstrate that statistically significant lesion-deficit mappings derived from mass-univariate analyses can be driven by only a small subset of the patients. Second, they constrain the nature of the interpretation that can be drawn. Specifically, we cannot conclude that a region is “necessary” for the lost function when the majority of patients with damage to the region do not have the deficit. Even in those that do have the deficit, we cannot exclude (without further analyses) that the deficit arose because these patients had large lesions that damaged other areas that are needed to support recovery (see Price et al., 2017 for more details on these arguments). Furthermore, Analysis 2 illustrates that by removing patients with large strokes and damage to regions identified in previous steps, we might have been able to unmask subtler effects that would have otherwise been missed. Future studies, however, will need to confirm the importance of potentially unexpected lesion-deficit relationships.

### 4.3 Damage limited to the identified regions may not be necessary or sufficient to cause the deficit

Analysis of the lesion sites in individual patients showed that all subjects with the deficit of interest following damage to the frontal, temporal and putamen regions had lesions that extend beyond the borders of these statistically-thresholded voxel-based regions (see Fig. 4). In this sense, the voxel-wise statistical analyses have been useful for pinpointing the brain areas where damage is associated with a deficit of interest but cannot give us the exact borders of the critical lesion sites. In the absence of other patients with focal damage to the regions of interest, we cannot establish whether damage to a region identified in a voxel-based analysis is sufficient to cause the deficit of interest. This illustrates the importance of including patients with focal lesions to determine whether the deficit of interest is the consequence of focal damage to the region of interest or much more extensive damage that includes the identified region.

### 4.4 The pros and cons of conducting voxel-based lesion analyses on binary compared to continuous measures of brain damage

Our study also showed that the same anatomical regions were identified irrespective of whether the analysis was conducted on binary lesion images (indexing the presence or absence of damage) or continuous measures of structural abnormality (e.g., the fuzzy lesion images generated from our automated lesion identification procedure). The extent of the effects was, however, larger when we used the continuous rather than binary measures of brain damage, while controlling for all other factors; and, even though the same patient sample was included in the statistical analyses (i.e. Analysis 1). This result suggests that images that encode the degree of abnormality on a continuous rather than binary scale (e.g. Price et al., 2010; Fridriksson et al., 2013) may increase sensitivity to lesion-deficit relationships perhaps by avoiding classification errors that are introduced when binary images are generated (manually or via automated procedures). Further studies are required, nonetheless, to determine how consistently this advantage is observed. On the other hand, continuous images may lack specificity and detect effects that are unrelated to the lesion but potentially caused by normal inter-subject variability.

In the same vein, care needs to be taken when reporting the results of analyses that use continuous measures of structural integrity. This is because significant structure-function relationships can be identified in healthy (undamaged) tissue (due to normal inter-subject variability). This is particularly important to consider given the numerous VBM studies of the undamaged brain that have shown more grey or white matter volume or density in those with higher abilities (e.g., Richardson and Price, 2009). Again the confound can be avoided by reporting (i) the incidence of damage to each of the identified regions; and (ii) the incidence and severity of a deficit of interest in the context of a lesion. In addition, in the current study, we limited our analysis to voxels that were damaged in at least 5 patients as indexed by the binary lesion images.

### 4.5 Implications for future lesion-deficit studies

Finally, in this study, we have shown how iterative analyses along with post-hoc tests can help to understand some of the limitations of mass-univariate techniques. Our results showed that removing patients with damage to regions identified in previous analyses improved the sensitivity to additional lesion-deficit associations, which increases the likelihood of successfully detecting other possible lesion sites that might impair a given cognitive function. The essential contribution of the current paper is thus to reveal a problem, that of susceptibility to false negatives, which we have demonstrated by adopting an iterative mass-univariate approach. Determining the optimal way to resolve this issue awaits further work. In particular, it is likely that there will be circumstances where the iterative procedure presented here will not be practical to use, and multivariate methods such as machine learning (Hope et al., 2013, Mah et al., 2014, Zhang et al., 2014) will be needed to map the different patterns of damage that can lead to impaired performance in the context of large-scale distributed networks. Accordingly, we plan to explore how these ideas could be integrated into a machine learning approach (such as decision-tree classification), while considering the possibility that such methods could over- or under-fit, with corresponding consequences for false positive rates.

Another important implication of our results is that they highlight how very large samples of patients who have, collectively, incurred a wide range of focal lesions are needed if we are to identify and pinpoint all the brain regions that are critical to outcome and recovery after stroke. This challenge is not limited to univariate voxel-based analyses but also applies to multivariate techniques. It calls for international collaborations and data sharing. For the time being, however, the results from smaller samples, with detailed post-hoc analyses can be used to: (i) identify regions where damage may sometimes result in a functional impairment; (ii) report the consistency of these effects in the available sample; and (iii) dissociate lesion sites that have effects on different functions.

Findings from the current study also indicate that the results from univariate voxel-based lesion-deficit mapping analyses can only be used to make inferences about outcome and recovery after stroke at the population level but cannot be utilised to make inferences at the individual level.

## 5 Conclusions

Overall, this study set out to show (i) how false negative results arise when the same deficit can be caused by different lesion sites and (ii) how false negative effects can be recovered using iterative voxel-based lesion analyses. In addition, our post-hoc tests have also demonstrated several other limitations of mass-univariate techniques including: (iii) statistically significant lesion-deficit mappings can be driven by a subset of patients; and (iv) the areas that reach statistical significance in voxel-based lesion-deficit mapping are much smaller than the true extent of the lesion causing the deficit. Although, it might be the case that only the significant part is causing the deficit, this needs to be demonstrated by identifying patients who only have damage to the critical part using a focal lesion approach. Without such a demonstration, we cannot exclude the possibility that voxel-based analyses are only revealing the tip of the iceberg.

Finally, future studies using multivariate approaches such as machine learning will be useful to overcome some of the issues illustrated here. However, multivariate techniques will not be enough; explanatory models of neural networks and degenerated pathways are also necessary. A promising avenue of research will be the integration of results from lesion analyses with those from functional imaging and connectivity studies, which will allow us in the future to gain a better understanding of the relationships between structure, function, and outcome in the damaged brain.
