# Supplementary material for: How distributed processing produces false negatives in voxel-based lesion-deficit analyses
Source: Neuropsychologia. 2018 Jul 1;115:124–33. doi: 10.1016/j.neuropsychologia.2018.02.025 (PMC6018567; doi:10.1016/j.neuropsychologia.2018.02.025)
Supplement: Supplementary file 1 — Supplementary material [file mmc1.pdf]

SUPPLEMENTARY MATERIAL

How distributed processing produces false negatives in voxel-based lesion-deficit analyses

Simulations of the two-phase analysis

To assess the validity of the two-phase analysis procedure used in this paper, we simulated null data sets and then assessed the false-positive behaviour of each of the phases. The simulation involved the following nine steps.

Methods

1) Set-up: the simulation has one independent variable (IV) and a sequence of dependent variables (DV) (we have explored including between 2 and 180 DVs). One could think of each DV as corresponding to a voxel, or ROI, with each value of the variable corresponding to a patient's damage score at that location. In order to assess the implications of the basic principles underlying the two-phase analysis, we simulated an abstract, canonical, formulation of the analysis, with each DV sampled independently from all others, and, of course, independently of the IV.

2) Simulation hypothesis: the null is simulated, so each of the variables (IV or DVs) is randomly sampled from a uniform distribution in the range [0,1].

3) First phase: each DV is correlated with the IV, generating one correlation for each DV. Each of these correlations is then tested for significance. Since DVs are independent, we Bonferroni correct to ensure a corrected alpha-level of 0.05. We call the DVs found significant in this first phase *sig DVs*, and those not significant *non-sig DVs*.

4) Entry criterion: If there is at least one sig DV, the algorithm enters the second phase.

5) Second phase selection: for each sig DV, the simulation finds entries in it that are above a pre-set cut-off. Each such entry corresponds to a particular participant's "damage" for that dependent variable.

6) Exclusion 1: exclude from IV and all DVs, all participants that cross the cut-off for sig DVs.

7) Exclusion 2: exclude all sig DVs. We call the variables resulting from this and the previous exclusion *filtered IV* and *filtered DVs*.

8) Correlations: the algorithm then calculates correlations between filtered IV and each filtered DV, and determines p-values for each of these correlations. This gives as many p-values as there are non-sig DVs.

9) Distributions: histograms are then generated for the following,

a) all p-values from 1st phase;

b) all p-values from 2nd phase, one for each non-sig DV.

Results

The key findings are 1) the 1st phase p-values distribution is uniform; while 2) the 2nd phase p-values distribution is most often not discernibly different from uniform, although for some parameter settings, there is a small *deflation* of false positives. This pattern becomes clear when the cut-off for exclusion is high, i.e. only a small number of participants are excluded, and there are few dependent variables in the model: less than ~20. **Figure S1** shows a typical 1st phase distribution; **Figure S2** shows the most typical 2nd phase distribution; and **Figure S3** shows an example of the parameter-specific deflation pattern for the 2nd phase distribution.

These results suggest that the two-phase procedure is well-behaved. In particular, there is no evidence of an *inflation* of false positives, which would manifest as a skewing of probability mass *towards* the <0.05 range. The *deflation* of false positives (i.e. a skewing *away* from the <0.05 range) we observe for the 2nd phase p-values, is firstly small, secondly, restricted to very specific regions of the parameter space and also not surprising. The non-sig DVs upon which the 2nd phase is focussed, have necessarily failed to be found significant in the 1st phase. Since the IV is the same in both phases, there will be a tendency for DVs not found significant to the IV in the 1st phase, also not to be found significant to the IV in the 2nd phase.

Although we are unable to be sure on the basis of the false positive tests run here (where we are simulating the null), this deflation raises the possibility that the two-phase procedure may incur a very slight reduction in statistical power in a specific region of parameter space. The region where this deflation obtains, though, i.e. a high cut-off threshold and few dependent variables, is not a likely target for the 2-phase analysis, where more than a few participants will be excluded when entering the 2nd phase and the number of dependent variables/voxels is likely to be extremely large.

Supplementary Figures

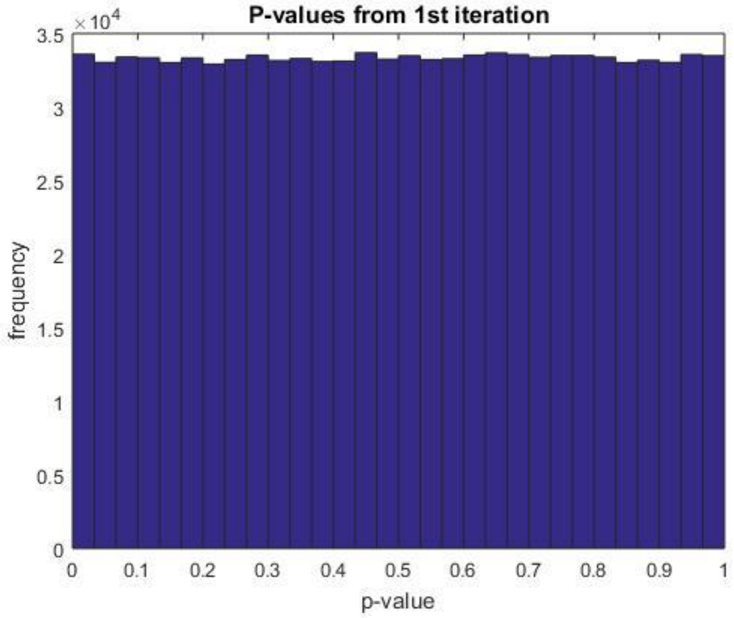

Figure S1: Distribution of p-values generated in a typical 1st phase of our simulations of the two-phase analysis. Since the simulations are under the null, a uniform distribution indicates a well-behaved test.

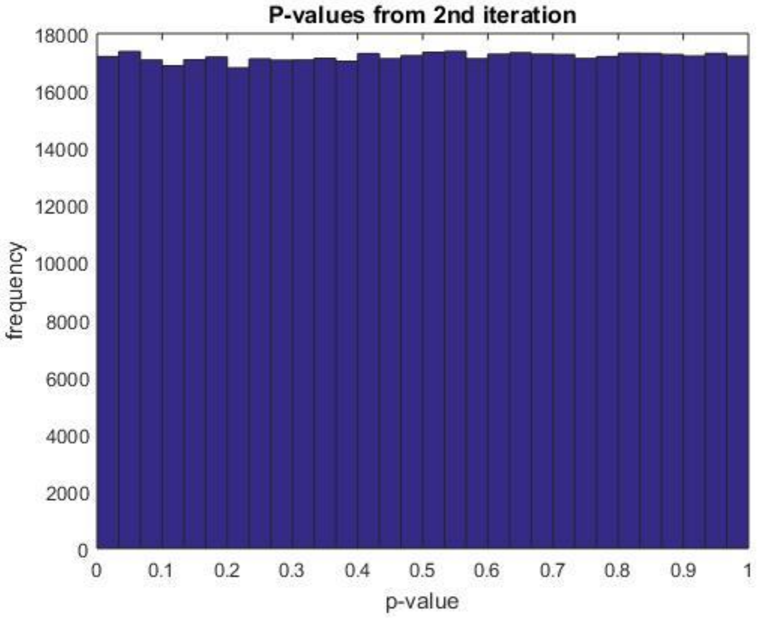

Figure S2: Distribution of p-values generated in a typical 2nd phase of our simulations of the two-phase analysis. Since the simulations are under the null, a uniform distribution indicates a well-behaved test.

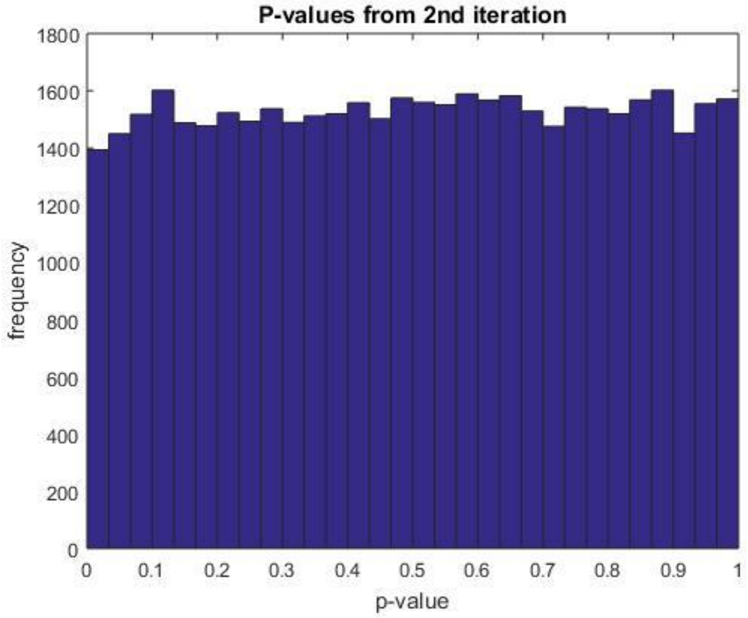

Figure S3: Distribution of p-values generated in a 2nd phase of our simulations of the two-phase analysis. This distribution indicates a very small, deflation of the false-positive rate. That is, probability mass is slightly reduced in the <0.05 range. This pattern is only evident in a very specific and small range of the parameter space: high cut-off and few dependent variables.
